# Antioxidant capacity of an ethanolic extract of *Elaeagnus* x *submacrophylla Servett*. leaves

**DOI:** 10.1016/j.heliyon.2024.e28067

**Published:** 2024-03-19

**Authors:** Hélène Bisi, Michel Bonnard, Laurianne Simon, Marie Morille, Sylvie Bégu, Isabelle Parrot

**Affiliations:** a IBMM, University of Montpellier, CNRS, ENSCM, 1919 Route de Mende, 34095, Montpellier, France; b FLORE SCOLA, 541 Rue des Vautes, 34980, Saint-Gély-du-Fesc, France; c ICGM, University of Montpellier, CNRS, ENSCM, 1919 Route de Mende, 34095, Montpellier, France

**Keywords:** *Elaeagnus x submacrophylla*, Leaves, Green extraction, Antioxidant capacity

## Abstract

In this study, we investigated the ethanolic extraction of the leaves of a very common but little studied plant species, *Elaeagnus* x *submacrophylla* Servett. and the opportunity of generating an antioxidant ingredient. The phytochemical profile of an ethanolic extract is also described here using gas chromatography and ultra-performance liquid chromatography, both combined with mass spectrometry (GC-MS and UPLC-MS), highlighting the presence of flavonoids, saponins, triterpenoids and a set of volatile compounds. Through *in vitro* assays (DPPH, ABTS, ORAC), the free radical scavenging capacity of the ingredient was then investigated (from 0.25 to 1.75 mmol TE/g) and compared with well-known standard antioxidants (BHT, gallic acid, quercetin, Trolox and vitamin C). In addition, *in cellulo* antioxidant capacity was performed using mice fibroblasts, revealing an activity equivalent to 50 mg/L of quercetin when tested the ethanolic extract in the concentration range of 50–300 mg/L, suggesting a synergistic combination effect of the identified phytochemicals. These results support the use of *Elaeagnus x submacrophylla* as a source of antioxidant ingredients.

## Graphical abstract

**Figure:**
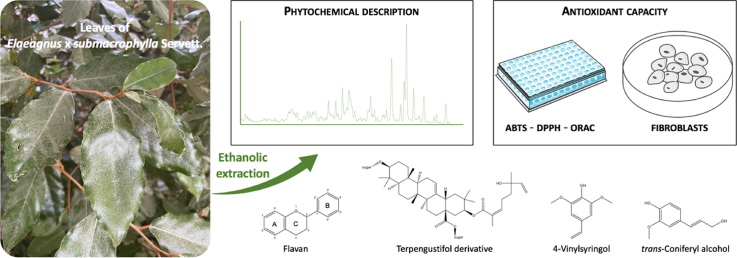

## 1 Introduction

Plants from the genius *Elaeagnus*, belonging to the Elaeagnaceae family which includes over 80 species, can be considered as a multipurpose source of bioactive phytoconstituents [1]. For example, *E. umbellata* (Autumn olive) is described in the literature as a shrub from the northwestern Himalayas, rich in bioactive compounds such as antioxidants, vitamins, minerals displaying high medicinal value [2,3]. For instance, berries, flowers, leaves and roots of this species have been employed for the treatment of asthma, pulmonary affections or myocardial infarction in folk uses [1]. Mention should also be made of flowers, seeds and barks of the Russian olive (*E. angustifolia*) which are traditionally used as herbal medicine with analgesic, antipyretic and diuretic properties due to the presence of alkaloids, carbohydrates, flavonoids, fatty acids, minerals, sterols and vitamins [1,4,5]. The preparation of extracts and bioactive fractions usually constitutes the raw materials of phytochemists for bioactivity assessment and chemical description of a given species. This methodology has been employed for *Elaeagnus* species, where solvent extracts have been employed for medicinal purposes such as anti-inflammatory, antioxidant, gastroprotective, wound healing or cardiovascular [4,6]. In some studies devoted to species of the genus *Elaeagnus*, particular bioactivities are directly related to presence of phytochemicals obtained by solvent extraction. This is the case of lycopene for example, a well-known antioxidant found in abundance in the edible fruits of *Elaeagnus* species, as well as linoleic acid and other phenolic acids (benzoic acid, cinnamic acid) and flavonoids (epigallocatechin gallate, myricetin), considered to mediate multiple health benefits (wound healing, cancer prevention, pain alleviation, etc.) [7]. Despite these medicinal and nutritional properties, plants from the genus *Elaeagnus* are still not available as healthcare products, and are still understudied. In addition to the described properties, the genus *Elaeagnus* could be easily cultived, particularly appropriate for permaculture [7], associated with recognized benefits in terms of increased carbon absorption. Usually used as ornamental species in urban areas, sometimes described as an invasive plant [8], *Elaeagnus* species remain underutilized despite the easy access to raw materials during pruning and shrub maintenance.

This general assessment is particularly manifest for the lesser-known *Elaeagnus* x *submacrophylla* Servett., widespread in the French Mediterranean region [9]. In terms of potential health benefits, this hybrid of *E. pungens* and *E. ebbingei* has only recently been studied for the antioxidant capacity and phytochemical composition of its floral extract using ethanol [10]. In order to extend this work, we investigated here the antioxidant capacity of an extract from leaves of *E. submacrophylla*. To obtain the plant extract, the leaves of this evergreen species were chosen, considering that they represent plant material accessible at any time of the year. Choosing to favor the use of ethanol as a green extraction solvent to prepare the extract, its phytochemical profile was also explored by gas chromatography and ultra-performance liquid chromatography, both combined with mass spectrometry. In order to compare the antioxidant potential of the foliar extract with the previously published floral extract, *in vitro* assays were conducted using the DPPH, ABTS and ORAC methods, in addition to the determination of the total phenolic content. To go further in the evaluation of the antioxidant capacity, *in cellulo* assays on the foliar extract were assessed on mice fibroblasts, after a cell viability determination. These cellular results were then correlated with the *in vitro* determination and the presence of bioactive compounds identified for the first time in the leaves of *E. submacrophylla*. To conclude, the data obtained in this study were compared with the phytoconstituents and antioxidant capacities of extracts from *Elaeagnus* species reported in the literature.

## 2 Results

### 2.1 Phytochemical profile

#### 2.1.1 Volatile compounds identified in EE

The GC-MS analysis of EE revealed the identification of sixty-nine volatile compounds (Table 1, Fig. S2) with major antioxidant compounds identified as phytol (no. 51, 15.79 ± 6.17%), a commonly found terpenoid, 4-vinylsyringol (no. 24, 10.38 ± 1.50%) and *trans*-coniferyl alcohol (no. 36, 8.22 ± 2.91%), two phenolic compounds. In terms of molecular families, the set of volatiles is represented by ∼39.1% of aromatic compounds (including 96.7% phenolic) and ∼60.9% of aliphatic compounds (including 32.8% terpenoids, 24.1% fatty acids, and 21.1% of carotenoid degradation products). Among the volatile compounds identified in leaves of plants of the genus *Elaeagnus* reported in the literature, only six compounds of the present study were previously reported: 2,4-dihydroxy-2,5-dimethyl-3(2H)-furanone (no. 2), 2-methoxy-4-vinylphenol (no. 16), hexahydrofarnesyl acetone (no. 40), palmitic acid (no. 44), phytol (no. 51) and probably the most widely antioxidant used, *α*-tocopherol (no. 66) [[11], [12], [13], [14]].

**Table 1:** Volatile compounds identified in the ethanolic extract of leaves of *E.* x *submacrophylla* using GC-MS.

No	RI exp	RI lit a	Compounds	Relative content (%) b
1	859	858	Furfuryl alcohol	0.14 ± 0.02
2	928	925	2-Hydroxycyclopent-2-en-1-one	0.08 ± 0.01
3	983	981	2,4-Dihydroxy-2,5-dimethyl-3(2H)-furanone	0.33 ± 0.06
4	997	1003	2H-Pyran-2,6(3H)-dione	0.13 ± 0.04
5	1027	1021	1-(1′-Pyrrolidinyl)-2-propanone	0.22 ± 0.06
6	1038	1033	Benzyl alcohol	0.25 ± 0.14
7	1056	1052	Furaneol	0.21 ± 0.10
8	1066	1069	2-Pyrrolidone	0.19 ± 0.15
9	1095	1090	*o*-Guaiacol	0.32 ± 0.22
10	1107	1110	6-Methyl-3,5-heptadien-2-one	0.09 ± 0.02
11	1147	1146	3-Hydroxy-2,3-dihydromaltol	1.38 ± 0.26
12	1218	1211	*p*-Vinylphenol	0.66 ± 0.13
13	1229	1226	5-Hydroxymethylfurfural	0.08 ± 0.08
14	1233	1238	2-Ethyl-3-methylmaleimide	0.29 ± 0.12
15	1264	1258	α-Ionene	0.15 ± 0.03
16	1321	1320	2-Methoxy-4-vinylphenol	2.66 ± 0.75
17	1357	1357	Syringol	0.56 ± 0.15
18	1391	1392	1-Tetradecene	0.38 ± 0.36
19	1406	1405	Vanillin	0.35 ± 0.22
20	1457	1457	*trans*-Isoeugenol	0.33 ± 0.25
21	1484	1427	Veratraldehyde	0.24 ± 0.08
22	1525	N/A^3^	2-Hydroxy-1-(1′-pyrrolidinyl)-1-buten-3-one	1.18 ± 0.09
23	1541	1541	Guaiacylacetone	0.32 ± 0.03
**24**	**1574**	**1571**	**4-Vinylsyringol**	**10.38 ± 1.50**
25	1593	1585	4,6,8-Megastigmatrien-3-one isomer #1	2.99 ± 0.16
26	1626	1617	3-Hydroxy-β-damascone	0.65 ± 0.23
27	1630	1631	4,6,8-Megastigmatrien-3-one isomer #2	1.57 ± 0.29
28	1637	1639	*p*-Coumaryl alcohol	0.33 ± 0.34
29	1643	N/A^3^	4,6,8-Megastigmatrien-3-one isomer #3	6.62 ± 1.49
30	1659	1660	Dihydroconiferyl alcohol	0.76 ± 0.16
31	1670	1667	Syringaldehyde	0.41 ± 0.10
32	1682	1680	*cis*-Coniferyl alcohol	0.59 ± 0.23
33	1709	1709	*trans*-4-Propenylsyringol	0.50 ± 0.25
34	1718	1710	Syringyl alcohol	0.49 ± 0.31
35	1740	1741	Dihydroconiferylic acid	0.56 ± 0.22
**36**	**1751**	**1743**	***trans*-Coniferyl alcohol**	**8.22 ± 2.91**
37	1757	1763	Myristic acid	0.24 ± 0.13
38	1781	1781	Syringyl acetone	0.23 ± 0.05
39	1787	1784	Loliolide	0.65 ± 0.07
40	1848	1848	Hexahydrofarnesyl acetone	4.57 ± 1.63
41	1902	1833	Dihydrosinapyl alcohol	0.29 ± 0.04
42	1912	1926	*trans*-3,4-Dimethoxycinnamic acid	0.08 ± 0.05
43	1926	1923	*cis*-Sinapyl alcohol	0.55 ± 0.02
44	1961	1960	Palmitic acid	5.56 ± 1.18
45	1981	1965	Methyl 2-(4-hydroxy-3,5-dimethoxyphenyl)acetate	0.44 ± 0.14
46	1994	1993	Ethyl palmitate	1.16 ± 0.35
47	2004	1981	*trans*-Sinapyl alcohol	4.09 ± 1.11
48	2060	2065	Margaric acid	0.23 ± 0.05
49	2100	2092	Methyl sinapate	0.13 ± 0.02
50	2106	2104	Methyl linolenate	0.13 ± 0.07
**51**	**2118**	**2116**	**Phytol**	**15.79 ± 6.17**
52	2136	2133	Linoleic acid	0.73 ± 0.11
53	2142	2138	Oleic acid	1.17 ± 0.08
54	2146	2141	Linolenic acid	5.44 ± 1.29
55	2162	2159	Stearic acid	1.15 ± 0.24
56	2171	2170	Ethyl oleate	0.18 ± 0.15
57	2173	2172	Ethyl linolenate	0.50 ± 0.33
58	2285	2284	Dimethylaminoethyl palmitate	1.13 ± 0.41
59	2458	2458	Dimethylaminoethyl linoleate	0.25 ± 0.05
60	2488	2486	Dimethylaminoethyl stearate	0.28 ± 0.05
61	2516	2498	2-Palmitoylglycerol	2.40 ± 0.67
62	2676	2669	*trans*-3,3′-Dimethoxy-4,4′-dihydroxystilbene	0.79 ± 0.30
63	2878	2860	α-Tocospiro A	1.13 ± 0.62
64	2902	2881	α-Tocospiro B	1.93 ± 0.51
65	2944	2932	4,4′-Dihydroxy-3,3′,5′-trimethoxystilbene	0.30 ± 0.01
66	3167	3167	α-Tocopherol	3.04 ± 0.09
67	3228	N/A c	Homoegonol	0.34 ± 0.10
68	3278	3290	Pinoresinol	0.16 ± 0.04
69	3525	3523	Medioresinol	0.32 ± 0.02

a Retention index reported from the NIST17 library.

b Mean relative content obtained with relative peak area percentage (TIC %), results are expressed as mean ± SD.

c Data not available in the NIST17 library.

#### 2.1.2 UPLC-MS-MS/MS

The UPLC-MS-MS/MS analysis of EE revealed the presence of ninety-six additional major compounds (thirty-three glycosylated flavonoids, thirty-nine saponins and twenty-four triterpenoids are proposed in Table S1). Among the group of flavonoids (Fig. S3), flavonols are only substituted by *O*-rhamnosyl and *O*-hexosyl groups. In addition, the major fragments identified by MS/MS show that the aglycone moieties of flavonoids are mainly composed of quercetin, kaempferol and isorhamnetin skeletons, as reported in the literature in the set of flavonoids extracted from flowers of *E.* x *submacrophylla* (including compounds no. 70–72, 79, 84, 85, 95, 96) [10]. Furthermore, additional flavonoids reported in the literature of phytoconstituents from plants of the genus *Elaeagnus* were also potentially identified in this study, such as astragalin (no. 90) [4], isorhamnetin-3-*O*-β-galactopyranoside (no. 91) [1] and tiliroside or regioisomeric derivatives (no. 95–97) [1]. Regarding the set of saponins (Fig. S3), terpengustifol A (C_69_H_110_O_29_, *m/z*_calc_ 1401.7055 [M − H]^-^), recently identified in flowers of *E*. *angustifolia* [15], is proposed in this study as compound no. 124 (*m/z*_obs_ 1401.7061 [M − H]^-^, Table S1). On the basis of its aglycone fragment ion observed at *m/z* 471.3475 [M − H]^-^, corresponding to C_30_H_47_O_4_^−^, a set of nine terpengustifol derivatives is also proposed (no. 102, 105–106, 113, 125, 131, 139–141, Table S1), all sharing the same aglycone fragment ion. In addition, compounds with molecular ions corresponding to the terpengustifol aglycone ion C_30_H_47_O_4_^−^ were also identified (no. 143–148, Table S1) as potential regioisomers or resulting from the fragmentation of higher terpengustifol saponins not detected in the 50–1500 *m/z* range. Besides, another potential sapogenin molecular ion was also detected at *m/z* 487.3405 [M − H]^-^, corresponding to C_30_H_47_O_5_^−^ (no. 142) and with fragment ions different from that of the terpengustifol sapogenins (*m/z* 443.3457, 427.3203 and 393.3144 respectively). The signal of this C_30_H_47_O_5_^−^ sapogenin ion, and/or its fragment ions, was identified among fragment ions of a set of twenty-nine saponins, proposed here under the trivial name of terpenmacrophylla derivatives (no. 99, 101, 107, 109–112, 114–123, 126–130, 132–138, Table S1). Compounds no. 109, 110 and 139 are proposed as terpenmacrophylla derivatives since their fragment ion at *m/z* 485.3255 [M − H]^-^, corresponding to C_30_H_45_O_5_^−^, can be represented as a structural equivalent of the terpengustifol sapogenin ion with an additional double bond. Due to the relative low molecular weight of compounds no. 120, 121, 123, 126, 127, 129 and 134 (<550 Da), their detection in the retention range of saponins certainly result from the fragmentation of higher terpenmacrophylla derivatives not detected in the 50–1500 *m/z* range. Compounds no. 111, 112, 114, 118, 122, 133, 136 and 140 are attributed here as terpenmacrophylla derivatives, since they share common fragment ions or fragmentation patterns with those detected for terpenmacrophylla sapogenin fragment ion(s). Moreover, the proposed identification of terpenmacrophylla derivatives constitutes the first identification of this group of saponins in leaves of *E.* x *submacrophylla*. Finally, the identification of a group of seventeen triterpenoids is proposed (no. 149–165, Table S1 and Fig. S3). Based on their molecular weight and fragmentation by MS/MS, four subgroups can be proposed, with compounds no. 149–151 as ursolic and oleanolic acids of coumaroyl (including regiosisomers), compounds 152–155 as acetylated triterpenoids, compounds 156–161 as *cis-* and *trans-*forms of caffeoeyltriterpenic acids and compounds 162–165 as *cis-* and *trans-*forms of 3-*O*-*p*-hydroxycinnamoyl ursolic and/or oleanolic acids. This proposed identification of triterpenoids is consistent with those identified in leaves of *E. rhamnoides* and *E. oldhamii* [1,16,17].

#### 2.1.3 Total phenolic content

In order to quantify the content of phenolic compounds highlighted by GC-MS and UPLC-MS-MS/MS, the Follin-Ciocalteu method was applied to EE. The results showed a total phenolic content (TPC) of 83.70 ± 4.35 mg GAE/g of EE, suggesting an interesting source of phenolic compounds such as those from the flavonoid group previously identified by UPLC-MS-MS/MS. The TPC of EE obtained from leaves of *E.* x *submacrophylla* is in the same order of magnitude than the ethanolic extract obtained from flowers of *E.* x *submacrophylla* (65.10 mg GAE/g) [10], the methanolic extract obtained from leaves of *E. latifolia* (61.15 mg GAE/g) [18] and the hydroethanolic extract obtained from leaves of *E. angustifolia* (65.35 mg GAE/g) [14].

### 2.2 Antioxidant potential

#### 2.2.1 Radical scavenging capacity

The antioxidant potential of EE was then assessed by its ability to scavenge free radicals. These results are presented in Table 2 and compared to pure antioxidant standards. As observed for the TPC determination, the radical scavenging capacity of EE is similar to that of the hydroethanolic extract obtained from leaves of *E. angustifolia* (TEAC_DPPH_ of 0.19 mmol TE/g and TEAC_ABTS_ of 0.29 mmol TE/g) [14] and the ethanolic extract of flowers of *E.* x *submacrophylla* (TEAC_DPPH_ of 0.40 mmol TE/g, TEAC_ABTS_ of 0.60 mmol TE/g and TEAC_ORAC_ of 1.30 mmol TE/g) [10], suggesting that both flowers and leaves of *E.* x *submacrophylla* may be combined for extraction or that leaves alone can substitute flowers as sources of natural antioxidants.

**Table 2:** Radical scavenging capacity of the ethanolic extract of leaves of *E.* x *submacrophylla* compared to pure antioxidant standards. Results are expressed as mean ± SD.

Samples	TEAC (mmol TE/g)		
	DPPH	ABTS	ORAC values
EE	0.25 ± 0.03	0.63 ± 0.08	1.75 ± 0.31
Gallic acid	14.47 ± 0.90	19.39 ± 3.77	Ranging from 6.2 ± 0.9 to 9.0 ± 1.0 a
Quercetin	7.86 ± 0.19	10.67 ± 0.82	22.23 ± 3.19
Vitamin C	6.52 ± 0.32	4.12 ± 0.98 b	Ranging from 1.9 ± 0.3 to 5.4 ± 0.1 a
BHT	1.24 ± 0.37	4.47 ± 0.24 b	Ranging from 0.73 ± 0.05 to 1.22 ± 0.04 a

a Data reported from the literature (gallic acid [[19], [20], [21]], vitamin C [19,20,22], BHT [[23], [24], [25]]).

b Not significantly different (*p* < 0.05).

#### 2.2.2 In cellulo antioxidant capacity

**Fig. 1:**
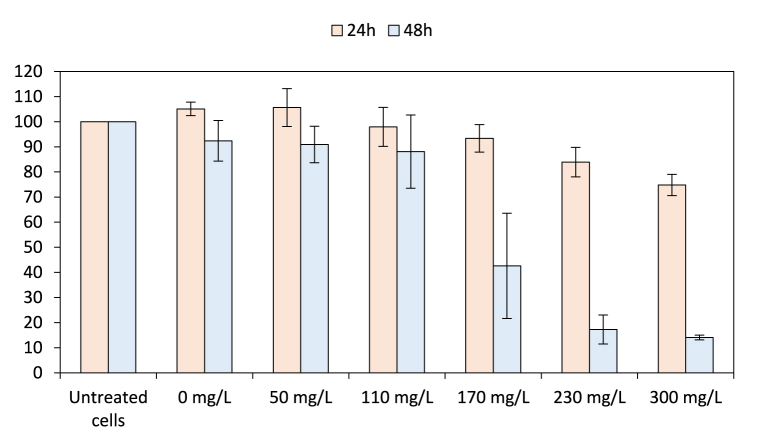
Cell viability determination of EE. The 0 mg/L concentration consisted of 2% ethanol. Untreated cells, considered equivalent to 100% cell viability, were used for results normalization (Student's *t*-test, differences were regarded as significant at the level *p* < 0.05).

In order to compare the previous *in vitro* free radicals scavenging capacity with the *in cellulo* antioxidant capacity of EE, cell-based assays were then conducted after determination of cell viability on a NIH/3T3 cell line (Fig. S4). The EE tested was considered to affect cell viability with cell viability below 70%, as recommended by ISO 10993–5:2009 [26]. After 24 h of treatment with EE, the tested concentrations range between 50 and 300 mg/L did not impact cell viability (105.64 ± 7.55%, 97.94 ± 7.76%, 93.39 ± 5.48%, 83.91 ± 5.89% and 74.80 ± 4.24%, respectively, Fig. 1). After 48 h of treatment, only concentrations of 50 and 110 mg/L did not affect the cell viability (90.93 ± 7.27%, 88.08 ± 14.59%, Fig. S4). Since the *in cellulo* antioxidant capacity is measured after 24 h of incubation, the concentration range of EE was set from 50 to 300 mg/L.

**Fig. 2:**
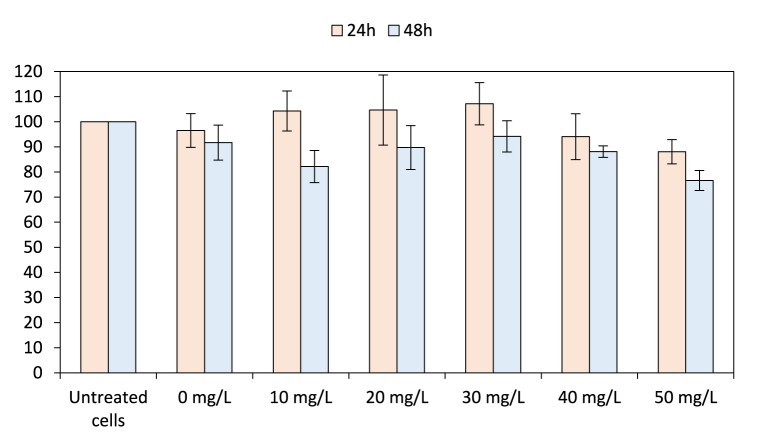
Cell viability determination of quercetin. The 0 mg/L concentration consisted of 2% ethanol. Untreated cells, considered equivalent to 100% cell viability, were used for results normalization (Student's *t*-test, differences were regarded as significant at the level *p* < 0.05).

In comparison, none of the tested concentrations of quercetin (10–50 mg/L) decreased significantly the cell viability after 24 h (104.29 ± 7.96%, 104.65 ± 13.95%, 107.18 ± 8.43%, 94.05 ± 9.12% and 88.05 ± 4.83%, respectively) and 48 h (82.16 ± 6.38%, 89.72 ± 8.74%, 94.17 ± 6.23%, 88.10 ± 2.28% and 76.61 ± 3.99%, respectively) of treatment (Fig. 2). Thus, the tested concentration of quercetin for the cellular antioxidant assay was set at 50 mg/L, the highest concentration limited by its solubility and enabling convenient comparison with the lowest EE concentration.

**Fig. 3:**
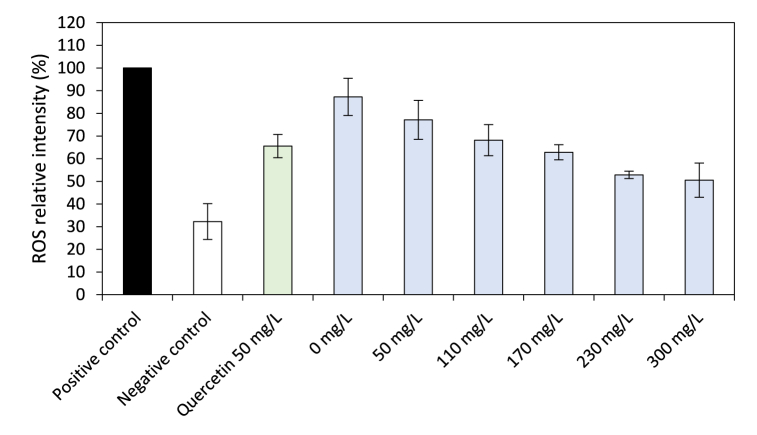
ROS relative intensity for both EE and quercetin. The 0 mg/L concentration consisted of 2% ethanol. Positive control, considered equivalent to 100% ROS intensity, was used for results normalization (Student's *t*-test, differences were regarded as significant at the level *p* < 0.05).

All the tested samples induced a significant decrease in ROS generation compared to the positive control except for the 2% ethanol sample (87.2 ± 8.2%, the 0 mg/L concentration, Fig. 3). Among the EE tested concentrations, only the 50 mg/L was not significantly different from the positive control (77.1 ± 8.6%). From 110 to 230 mg/L, the ROS intensity decreased in a dose dependent manner (68.1 ± 6.9%, 62.8 ± 3.3% and 52.8 ± 1.6%, respectively). At 300 mg/L, ROS intensity (50.5 ± 7.5%) was not significantly different from that at 230 mg/L. Taking into account the non-zero ROS relative intensity of the negative control, approximately two-thirds of the stress-generated intracellular ROS were quenched at the two highest EE concentrations. Quercetin tested here at 50 mg/L allowed to reduce one-thirds of ROS level (65.6 ± 5.1%). Compared to EE, ROS intensity with quercetin cannot be considered different from that of EE at concentrations 50–170 mg/L and significantly higher from that of EE at concentrations 230–300 mg/L. Thus, these two later concentrations have a slightly higher *in cellulo* antioxidant capacity than quercetin at 50 mg/L.

## 3 Discussion

In this study, the antioxidant capacity of an ethanolic extract (EE) of leaves of *E.* x *submacrophylla* was investigated for the first time. Promising results were obtained by *in vitro* assays when compared to well-known antioxidant standards, especially BHT, a synthetic antioxidant largely used in industry, as most results are around one order of magnitude difference. Among data reported in the literature for foliar extracts of plants in the genus *Elaeagnus*, the radical scavenging capacities are of the same order of magnitude as those obtained in this study. This is particularly relevant for the floral extract of *E.* x *submacrophylla* [10], thus enabling to consider substituting the collection of flowers by leaves, whose seasonality of collection is more extended in the perspective of producing an antioxidant ingredient. Focusing on other well-known natural antioxidants, these TEAC results are also comparable to red wine, tea, rosé wine and yerba mate. Indeed, considering the DPPH assay, 1 g of EE is equivalent to 30–40 mL of red wine [[27], [28], [29], [30], [31]], 235–295 mL of rosé wine [27,28,31], 75–90 mL of green tea hot infusion [[32], [33], [34], [35], [36], [37]] and 0.3–0.4 g of yerba mate hydroalcoholic extract [[38], [39], [40]]. Regarding the ABTS assay, 1 g of EE is equivalent to 26–57 mL of red wine, approximately 240 mL of rosé wine, 35–40 mL of green tea hot infusion, 1.5–1.6 g of yerba mate hydroalcoholic extract and 180–200 mL of yerba mate infusion [[27], [28], [29], [30], [31], [32], [33], [34], [35], [36], [37], [38],^41^]. Finally, the ORAC values of 1 g of EE is equivalent to 35–150 mL of red wine, 156–194 mL of rosé wine, 21–83 mL of green tea hot infusion, 65–87 mL of yerba mate infusion and 0.7–0.8 g of yerba mate hydroalcoholic extract [[27], [28], [29], [30], [31], [32], [33], [34], [35], [36], [37], [38], [39], [40], [41]]. These data are consistent with the presence of various phenolic compounds, as determined by the Follin-Ciocalteu method, representing 83.70 ± 4.35 mg GAE/g of EE and most notably with the identification of glycosylated flavonoids, such as astragalin, identified here for the first time in *E.* x *submacrophylla*. Known as kaempferol-3-*O*-glucoside, this flavonol has a large spectrum of pharmacological activities, including antioxidant, cardioprotective and anti-inflammatory [42]. Furthermore, we cannot exclude that less abundant compounds or compounds not detected by gas or liquid chromatography may also contribute to the antioxidant activity of EE. A bio-guided fractionation of EE would enable to assign the antioxidant capacity to a specific or reduced set of compounds (known or new). In a synergistic combination as EE or as fractions or isolated compounds from EE, saponins, terpenoids, astragalin, flavonoids and other undetected compounds could potentially provide a therapeutic or nutritional solution against various diseases, which deserves to be further investigated. At the present state of the knowledge, the antioxidant capacity of EE could be potentially enhanced by including a hydrolysis of flavonoids during the preparation of EE since aglycone flavonoids are recognized as more active than their glycosylated forms [43].

In a desire to compared *in vitro* with *in cellulo* data, the antioxidant capacity of EE of *E.* x *submacrophylla* was also tested here on mice fibroblasts. Since *in cellulo* assays are the intermediate step between *in vitro* and *in vivo* trials, the promising observed antioxidant capacity at concentrations ranging from 50 to 300 mg/L supports the use of EE as an antioxidant ingredient. In addition, an equivalent antioxidant capacity with 50 mg/L of quercetin was observed from 50 to 170 mg/L of EE and higher at 230 and 300 mg/L, suggesting a potential synergetic effect *in cellulo* of flavonoids, saponins, triterpenoids and volatiles among the phytochemical set of compounds in EE, since *in vitro* trials have shown a lower antioxidant capacity than that observed for quercetin. The results presented here obtained in year N were duplicated in year N+1, demonstrating similar antioxidant capacity (Table S2), and suggesting significant reproducibility in extract production from one year to the next.

## 4 Conclusion

In recent decades, when the search for bioactive compounds has taken on considerable importance, it has become essential in the field of extraction for the production of natural ingredients, to provide a global eco-responsible solution, from the plant material to its extraction method. Due to its widespread distribution, described in some countries as invasive, its many non-deciduous leaves, its low water requirements, high resistance in temperate environments and its great tolerance to pruning, *E.* x *submacrophylla* is a shrub that deserves to be more widely studied. The work presented in this article reveals, in particular, the importance of this lesser-known medicinal plant as a potential source of antioxidants for potential pharmaceutical, nutritional or cosmetic applications. While the focus of our work was exclusively on antioxidant effect in relation to potentially active phytoconstituents such as flavonoids, saponins, triterpenoids and more generally phenolic compounds, this set of compounds identified here in the ethanolic extract of *E.* x *submacrophylla* points to benefit in other areas, in particular for anti-inflammatory activities, neuroprotective or anticancer properties. Future work dedicated to the precise structural description of a whole new family of molecules, called here terpenmacrophylla derivatives, may also provide after separation and study of structure-activity relationships, access to other specific biological activities.

## 5 Materials and methods

### 5.1 General experimental procedures

Chemicals and reagents were used without further purification. 2,2-azinobis(3-ethylbenzothiazoline-6-sulfonic acid) (ABTS), 2,2-azobis(2-methylpropionamide) dihydrochloride (AAPH), butylated hydroxytoluene (BHT), *n*-alkanes standard solutions (C7–C30 and C7–C40, 1000 μg/mL in hexane), fluorescein sodium salt, 6-hydroxy-2,5,7,8-tetramethylchroman-2-carboxylic acid (Trolox), acetonitrile (HPLC grade, >99.5%), quercetin hydrate, potassium persulfate, 2,2-diphenyl-1-picrylhydrazyl (DPPH^•^), anhydrous l-ascorbic acid and the Folin-Ciocalteu reagent (2 M) were purchased from Sigma-Aldrich (St. Louis, MO). Ethanol (99.8%) was purchased from VWR (Darlstadt, Germany). Gallic acid (anhydrous) was obtained from Merck (Darmstadt, Germany). Ultrapure water (0.055 μS/cm) was obtained with a Labostar ProTWF from Evoqua (Chaville, France). 2,7-dichlorofluorescein diacetate (DCFDA) and *tert*-butyl hydroperoxide solution (70% in water, TBHP) were obtained from Sigma-Aldrich (Germany). Sodium phosphate dibasic dodecahydrate and sodium phosphate dibasic dehydrates were obtained from VWR (Leuvin, Belgium). Formic acid (ULC/MS grade 99%) was obtained from Biosolve (Netherlands). Helium (5.6 purity) was obtained from Linde Gas (Saint-Priest, France). GC-MS analysis were conducted on a TRACE 1300 apparatus equipped with a DSQ II single quadrupole spectrometer (Thermo Fisher Scientific, Waltham, MA) at an electron impact ionization of 70 eV. Recording of MS data was conducted in the range of *m/z* 33–650 amu at 6 scans/sec. Xcalibur 3.0.63 (Thermo Fisher Scientific) was used for recording and analyzing chromatograms. Experiments were conducted with a non-polar stationary phase (TG-5MS, 30 m × 0.25 mm i.d. x 0.25 μm, Thermo Fisher Scientific). The oven was set at 70 °C for 3 min, followed by a ramp to 250 °C at 10 °C/min, kept at 250 °C for 3 min and ramped again to 330 °C at 25 °C/min and finally kept at 330 °C for 3 min. The injector was set at 250 °C. The transfer line was kept at 350 °C and the MS ion source at 250 °C. Helium was maintained at 0.9 mL/min. EE solubilized in ethanol was injected in split ratio (1:10) and the Kováts retention index (RI) was calculated using the *n*-alkanes standards. The identification of compounds was conducted according to their mass spectra and RI compared to those reported in the library of the laboratory and online databases (the National Institute of Standards and Technology and the Adams database [44]). Relative content was obtained by peak area normalization with relative response factors take as one. Experiments were conducted in triplicate and results are expressed as mean ± standard deviation. UPLC-MS-MS/MS analyses were conducted on an Acquity H-Class UPLC (Waters, Milford, MA) combined with a quadrupole time-of-flight spectrometer. The blank (EtOH) and EE (solubilized in EtOH) were injected at 0.5 μL. The reverse stationary phase used in this study was a Kinetex EVO C18 (100 Å; 150 mm × 2.1 mm x 2.6 μm; Phenomenex, Torrance, CA) kept at 25 °C. The mobile phase consisted of ultrapure water (solvent A) and acetonitrile (solvent B), both acidified with formic acid (0.1%). Separation was conducted at 0.5 mL/min with a linear gradient from 0 to 100% solvent B for 120 min. Mass detection was conducted in negative ionization mode (source temperature set at 140 °C, capillary voltage of 2.5 kV and cone voltage of 40 V, nitrogen desolvatation gas flow set at 1000 L/h, desolvatation temperature set at 450 °C) with a HDMS Synapt G2-S (Waters). Acquisition of the *m/z* range was set at 50–1500. MS/MS was performed with argon as the collision gas at 30 eV (collision energy). MassLynx 4.2 (Waters) was used for data acquisition and processing. For the determination of the total phenolic content using the Folin-Ciocalteu method, EE and gallic acid were both dissolved in 75/25 (V/V) EtOH/H_2_O. Samples (100 μL of EE, gallic acid or solvent as blank) were mixed with the Folin-Ciocalteu reagent (500 μL) followed by the addition, after 5 min, of 75 g/L Na_2_CO_3_ (400 μL). Samples were kept at 20 °C in the dark for 2 h and the absorbance was then read with a UV-1800 spectrophotometer (Shimadzu, Marne-la-Vallée, France) at 750 nm. Experiments were performed three times and results are expressed as mg of gallic acid equivalent (GAE)/g of foliar EE (mean ± standard deviation).

### 5.2 Plant material

Leaves of *E. submacrophylla* were collected from ornamental shrubs in the autumn season in Montpellier, France (coordinates 43° 28′ 5.1″ N and 3° 51’ 36.6” E, altitude of 43 m, average annual precipitation 739 mm, average annual temperature 15.0 °C). Botanical identification was conducted by Dr. Yves Caraglio, a local botanist. Voucher specimens were deposited at the herbarium of the University of Montpellier, France (MPU814005). Fresh leaves were washed with demineralized water in order to remove dust and exogenous contaminants, and were finally air-dried. Clean and dry leaves were immediately subject to extraction with absolute ethanol.

### 5.3 Ethanolic extraction

Leaves were immersed in absolute ethanol and progressively cut into pieces of approximately 5 mm using a hand blender. Absolute ethanol was preferred to aqueous ethanol in order to allow a comparison with the floral extract obtained in our previous work [10], as well as to enable the extraction of volatile and less polar compounds than those that can be extracted with aqueous ethanol. Additional absolute ethanol was then added to pieces of leave immersed in ethanol (final ratio of 1:3 w/w) and kept under constant stirring for 24 h at 20 °C. Ethanol was then filtrated on cotton wool and evaporated under reduced pressure at 25 °C, giving a dark-green extract (extraction yield of approximately 2.0 wt%). The dark-green extract was treated with boiling absolute ethanol and kept under reflux. This operation was repeated until the complete ethanol discoloration (four times at least). Each fraction was filtered on cotton wool, pooled together and stored overnight at 4 °C. Ethanol was then filtrated at 4 °C in order to remove non-soluble materials. Finally, ethanol was removed under reduced pressure at 25 °C, leading to the final ethanolic extract (EE, extraction yield of approximately 0.6 wt% of the initial fresh leaves). The extraction yields of EE were calculated according to:

Yield(wt.%)=(massofEE/massoffreshleaves)X100

### 5.4 Antioxidant activity

#### 5.4.1 DPPH radical scavenging capacity

The scavenging capacity of EE and various standards (including Trolox, BHT, ascorbic acid, quercetin and gallic acid) was assessed by using the methodology outlined by Bendaikha et al. [45] with minor modifications as detailed by Parrot et al. [10]. Samples prepared in ethanol (50 μL) were mixed with the DPPH^•^ solution at 75 μM (950 μL). Samples were kept for 30 min in the dark. The absorbance was measured at 515 nm with a UV-1800 spectrophotometer. A blank (ethanol) was prepared for each sample. The inhibition percentage was determined according to:

Inhibition%=[(Acontrol−Asample)/Acontrol]×100

The 50% inhibition concentration of samples (IC_50_ value) was determined thanks to the calibration curve inhibition % = f(concentration). Experiments were performed three times. Results of the standard calibration curves can be find in Parrot et al. [10]. The TEAC (Trolox Equivalent Antioxidant Capacity) of samples were determined using:

TEAC(mmolTE/g)=IC50(Trolox)/IC50(standards/EE)(g/L),withIC50(Trolox)=0.0233mM.

Results are expressed as mean ± standard deviation.

#### 5.4.2 ABTS radical scavenging capacity

The methodology of Re et al. [46] was used with minor modifications as detailed by Parrot et al. [10]. The stock solution of ABTS^•+^ was prepared by mixing an equal volume of aqueous K_2_S_2_O_8_ (2.45 mM) and ABTS (6.57 mM) and was kept at 20 °C for 16 h in the dark. The working solution was then obtained by dilution of the previous solution with ethanol until an absorbance, at 752 nm, of 0.84 ± 0.02. Dilutions of samples (EE and standards) were prepared in ethanol (20 μL of sample and 980 μL of the working solution). The negative control was prepared with 20 μL of ethanol. The blank was prepared by mixing H_2_O and EtOH (20/980, V/V). After incubation in the dark for 30 min, samples absorbance (including the negative control), was read with a UV-1800 spectrophotometer at 752 nm. Experiments were performed three times. The absorbance was used to calculate the TEAC, the IC_50_ and the ABTS^•+^ inhibition according to the method previously employed for the determination of the DPPH radical scavenging capacity. Results of the standard calibration curves can be find in Parrot et al. [10]. The TEAC of samples were determined using:

$$\text{TEAC}\;\left(\text{mmol}\;\text{TE}/g\right)={\text{IC}}_{50\left(\text{Trolox}\right)}/{\text{IC}}_{50\left(\text{standards}/\text{EE}\right)}\;\left(g/L\right),\text{with}\;{\text{IC}}_{50\left(\text{Trolox}\right)}=0.01237\;\text{mM}.$$

Results are expressed as mean ± standard deviation.

#### 5.4.3 Oxygen radical absorbance capacity (ORAC)

The ORAC assay was conducted using the methodology of Dudonné et al. [47]. The fluorescein and AAPH solutions were prepared with a 75 mM sodium phosphate buffer at pH 7.4 (final concentrations in the cuvettes of 12 mM and 70 nM, respectively). The fluorescein stock solution (0.85 mM) was stored at 4 °C and used to prepare fresh working solutions. Samples (Trolox, quercetin and EE) were prepared in EtOH/75 mM buffer (25:75, V/V). Samples (300 μL) were mixed with the fluorescein stock solution (1.8 mL) and incubated 5 min at 37 °C. The addition of AAPH (900 μL) started the reaction. The fluorescence intensity was read every for 45 min 30 s (*λ* excitation at 493 nm, *λ* emission at 511 nm) with a J-815 CD spectrometer (JASCO, Lisses, France) equipped with a fluorescence monochromator, a Peltier temperature controller and a path length of 1 cm. Spectrometer parameters were set as: digital integration time of 1 s, excitation and emission bandwidth of 10 nm, fluorescence detector voltage of 700 V and the temperature was kept at 37 °C. The blank (300 μL) was prepared by mixing EtOH and the 75 mM sodium phosphate buffer (25:75, V/V). The AAPH and fluorescein solution was prepared and measured every day. Experiments were performed three times. The AUC (area under the curve) was measured by integration using:

$$AUC=\sum_{i=1}^{i=90}\frac{\left(t_{i+1}-\;t_{i}\right)*\left(f_{i+1}+f_{i}\right)}{2}$$

where t_i_ is the fluorescence intensity and the time and at reading i. The net AUC was obtained by subtracting the AUC of samples to that of the blank. The ORAC values were expressed in mmol TE/g quercetin or EE according to the following equation:

ORAC value = equivalent Trolox concentration/sample concentration (mean ± standard deviation). Results are expressed as mean ± standard deviation.

#### 5.4.4 In cellulo antioxidant capacity

The mice fibroblasts cell line used in this study (NIH/3T3) was purchased from the American Type Culture Collection (Rockville, MD, USA). Cells were maintained in Dulbecco's Modified Eagle Medium (DMEM, Gibco) with phenol red and supplemented with 10% fetal bovine serum (V/V), 1% penicillin and streptomycin (V/V, final concentration of 100 U/mL and 100 μg/mL, respectively) and 1% l-glutamine (V/V, final concentration of 2 mM). Cells were incubated at 37 °C with 5% CO_2_ atmosphere. For sub-culturing, cells were detached every 3–4 days using trypsin and were suspended in culture medium before plating at 4–5.10^3^ cells/cm^2^. Cells were observed with an EVOS microscope AMF4300 (Fig. S1, Life Technologies, USA). For cell viability determination, the MTS (3-(4,5-dimethylthiazol-2-yl)-5-(3-carboxymethoxyphenyl)2-(4-sulfophenyl)-2H-tetrazolium) assay, based on the reduction of a tetrazolium salt to a red/purple formazan compound (*λ* = 490 nm), was employed. A total of 7500 cells/well were seeded in a 96-well plate and were incubated 24 h at 37 °C and 5% CO_2_. Stock solution of quercetin (2.5 mg/mL) and EE (15 mg/mL) were prepared in EtOH and filtered with PTFE 0.2 μm filter. Quercetin and EE samples were respectively diluted from 50 to 10 mg/L and 300 to 50 mg/L in EtOH and DMEM to ensure 4% ethanolic solutions. After adhesion, cells were treated with quercetin, EE and EtOH as a control (2% in well). Negative control consisted of untreated cells. After 24 and 48 h of incubation, a CellTiter 96® aqueous non-radioactive cell proliferation assay (Promega) was added to each well followed by 2 h of incubation at 37 °C and 5% CO_2_. The absorbance was measured at 490 nm, in triplicate, using a Multiskan GO microplate spectrophotometer (Thermo Fisher Scientific). The cellular viability was determined after normalization to untreated cells representing 100% viability (cellular viability = A_sample_/A_negative control_). Experiments were performed in triplicate. The cell viability data were analyzed using Student's *t*-test, and differences were considered significant at *p* < 0.05. Results are expressed as mean ± standard deviation.

The *in cellulo* antioxidant capacity assay was adapted from the method employed by Moine et al. [48] and Simon et al. [49]. After incubation of cells (20,000 cells/cm^2^ in a 96 well black plate with clear bottom, Corning® Massachusetts, USA) with samples for 24 h, cells were washed twice with PBS and 200 μL of the DCFDA reagent was added (10 μM in DMEM without phenol red, supplemented with 1% FBS). After incubation at 37 °C, 5% CO_2_ for 30 min, cells were washed with PBS and 200 μL of DMEM (without phenol red), supplemented with 1% FBS. TBHP (*tert*-butyl hydroperoxide) was added (10 μL/well, final concentration of 0.5 mM) and cells were incubated for another 30 min. Fluorescence was measured at 535 nm (*λ* excitation at 485 nm, 0.1 s counting time, 8 W lap energy) with a Tristar LB941 spectrofluorometer (Berthold Technologies, Bad Wildbad, Germany). Samples alone did not emit fluorescence at *λ*_ex_ 485 nm/*λ*_em_ 535 nm. Experiments were performed in triplicate. The radical oxygen species (ROS) relative intensity was determined after normalization to the positive control representing 100% ROS intensity (ROS relative intensity = Fluorescence intensity_sample_/Fluorescence intensity_positive control_). The *in cellulo* antioxidant capacity data were analyzed using Student's *t*-test, and differences were considered significant at *p* < 0.05. Results are expressed as mean ± standard deviation.

## Data availability statement

Original data of this study are available on request.

## CRediT authorship contribution statement

**Hélène Bisi:** Writing – review & editing, Writing – original draft, Validation, Investigation, Funding acquisition, Formal analysis, Data curation, Conceptualization. **Michel Bonnard:** Writing – review & editing, Writing – original draft, Validation, Investigation, Funding acquisition, Formal analysis, Data curation, Conceptualization. **Laurianne Simon:** Writing – review & editing, Writing – original draft, Validation, Investigation, Funding acquisition, Formal analysis, Data curation, Conceptualization. **Marie Morille:** Writing – review & editing, Writing – original draft, Validation, Investigation, Funding acquisition, Formal analysis, Data curation, Conceptualization. **Sylvie Bégu:** Writing – review & editing, Writing – original draft, Validation, Investigation, Funding acquisition, Formal analysis, Data curation, Conceptualization. **Isabelle Parrot:** Writing – original draft, Validation, Investigation, Funding acquisition, Formal analysis, Data curation, Conceptualization.

## Declaration of competing interest

The authors declare that they have no known competing financial interests or personal relationships that could have appeared to influence the work reported in this paper.
