# Supplementary material for: Antioxidant capacity of an ethanolic extract of Elaeagnus x submacrophylla Servett. leaves
Source: Heliyon. 2024 Mar 19;10(7):e28067. doi: 10.1016/j.heliyon.2024.e28067 (PMC10981013; doi:10.1016/j.heliyon.2024.e28067)
Supplement: Multimedia component 1 [file mmc1.docx]

Supplementary Materials for

Antioxidant capacity of an ethanolic extract of *Elaeagnus* x *submacrophylla* Servett. leaves

|  |
| --- |

**Table S1**

Proposed identification of phytochemicals from EE analyzed by UPLC-MS-MS/MS.

| **No.** | **RT (min)** | **Compounds** | **Molecular formula** | **Calc m/z**  **[M-H]^-^** | **Obs m/z**  **[M-H]^-^** | **Major fragments** |
| --- | --- | --- | --- | --- | --- | --- |
| 70 | 16.94 | Quercetin di *O*-hexosyl-*O*-rhamnoside | C_39_H_50_O_25_ | 917.2563 | 917.2516 | 446.0838, 299.0175, 271.0246 |
| 71 | 17.10 | Quercetin-*O*-hexosyl-*O*-hexosyl-*O*-rhamnoside (I) | C_33_H_40_O_21_ | 771.1984 | 771.1948 | 446.0838, 299.0175, 271.0246, 255.0279, 243.0285 |
| 72 | 17.45 | Quercetin-*O*-rhamnosyl-*O*-rhamnosyl-*O*-hexoside (I) | C_33_H_40_O_20_ | 755.2035 | 755.2049 | 446.0838, 299.0175, 271.0246, 199.0399 |
| 73 | 17.86 | Kaempferol di *O*-hexosyl-*O*-rhamnoside | C_39_H_50_O_24_ | 901.2614 | 901.2618 | 755.2049, 430.0856, 284.0307, 255.0314, 227.0363 |
| 74 | 17.94 | Kaempferol-*O*-rhamnosyl-*O*-hexosyl-*O*-rhamnoside (I) | C_33_H_40_O_19_ | 739.2086 | 739.2102 | 593.1526, 430.0856, 283.0223, 255.0279, 227.0331 |
| 75 | 18.10 | Kaempferol-*O*-hexosyl-*O*-hexosyl-*O*-rhamnoside | C_33_H_40_O_20_ | 755.2035 | 755.2049 | 430.0901, 283.0259, 255.0279, 227.0331 |
| 76 | 18.37 | Kaempferol tetra *O*-rhamnoside | C_39_H_50_O_23_ | 885.2665 | 885.2630 | 739.2102, 430.0901, 284.0307, 255.0279, 227.0361 |
| 77 | 18.37 | Kaempferol-*O*-hexosyl-*O*-rhamnosyl-*O*-rhamnoside | C_33_H_40_O_19_ | 739.2086 | 739.2102 | 430.0901, 283.0223, 255.0279, 227.0331 |
| 78 | 18.80 | Isorhamnetin-*O*-rhamnosyl-*O*-hexosyl-*O*-rhamnoside | C_34_H_42_O_21_ | 785.2140 | 785.2131 | 639.1603, 460.0974, 445.0779, 313.0346, 299.0175, 285.0410, 271.0246, 242.0209 |
| 79 | 19.03 | Rutin or regioisomeric derivative (I) | C_27_H_30_O_16_ | 609.1456 | 609.1443 | 446.0838, 299.0175, 271.0246, 255.0314 |
| 80 | 19.23 | Kaempferol-*O*-rhamnosyl-*O*-hexosyl-*O*-hexosyl-*O*-hexoside | C_42_H_46_O_23_ | 917.2352 | 917.2318 | 772.1785, 446.0838, 299.0213, 284.0307, 271.0246, 255.0279 |
| 81 | 19.43 | Kaempferol tri *O*-rhamnoside | C_44_H_50_O_24_ | 961.2614 | 961.2607 | 815.2067, 609.1497, 430.0901, 283.0259, 255.0279, 227.0331, 190.0256, 151.0035 |
| 82 | 19.58 | Quercetin-*O*-hexosyl-*O*-rhamnosyl-*O*-rhamnoside | C_33_H_40_O_20_ | 755.2035 | 755.2049 | 446.0838, 354.1106, 339.0833, 299.0175, 284.0307, 271.0246, 255.0279, 227.0331 |
| 83 | 20.16 | Rutin or regioisomeric derivative (II) | C_27_H_30_O_16_ | 609.1456 | 609.1443 | 300.0276, 271.0246, 255.0279, 243.0285, 227.0331 |
| 84 | 20.16 | Kaempferol-*O*-hexosyl-*O*-rhamnoside (I) | C_27_H_30_O_15_ | 593.1542 | 593.1526 | 430.0947, 283.0223, 255.0279, 227.0363 |
| 85 | 20.32 | Kaempferol-*O*-rhamnosyl-*O*-hexosyl-*O*-rhamnoside (II) | C_33_H_40_O_19_ | 739.2086 | 739.2042 | 593.1473, 430.0856, 283.0223, 255.0279, 227.0331 |
| 86 | 20.95 | Kaempferol-*O*-hexosyl-*O*-rhamnoside (II) | C_27_H_30_O_15_ | 593.1542 | 593.1526 | 430.0901, 283.0223, 255.0279, 227.0331 |
| 87 | 21.55 | Kaempferol-*O*-hexosyl-*O*-rhamnoside (III) | C_27_H_30_O_15_ | 593.1542 | 593.1526 | 284.0307, 255.0279, 227.0331 |
| 88 | 21.91 | Kaempferol-*O*-hexosyl-*O*-rhamnoside (IV) | C_27_H_30_O_15_ | 593.1542 | 593.1526 | 284.0307, 255.0279, 227.0331 |
| 89 | 22.87 | Rutin or regioisomeric derivative (III) | C_27_H_30_O_16_ | 609.1456 | 609.1443 | 327.1197, 314.0410, 299.0175, 271.0210 |
| 90 | 23.07 | Astragalin | C_21_H_20_O_11_ | 447.0927 | 447.0908 | 284.0307, 255.0279, 227.0331 |
| 91 | 23.83 | Isorhamnetin-3-*O*-β-galactopyranoside | C_22_H_22_O_12_ | 477.1033 | 477.1023 | 314.0410, 299.0213, 285.0373, 271.0246, 257.0460, 243.0285 |
| 92 | 30.03 | Quercetin-*O*-hexosyl-*O*-rhamnoside | C_23_H_28_O_19_ | 607.1147 | 607.1160 | 299.0554, 284.0307, 255.0279, 227.0331 |
| 93 | 30.72 | Kaempferol-*O*-rhamnosyl-*O*-hexosyl-*O*-hexosyl-*O*-rhamnoside (I) | C_43_H_54_O_21_ | 905.3079 | 905.3086 | 759.2469, 593.1419, 430.0901, 284.0307, 255.0314, 227.0331 |
| 94 | 30.72 | Isorhamnetin-*O*-hexosyl-*O*-rhamnoside | C_29_H_34_O_16_ | 637.1769 | 637.1780 | 329.0677, 314.0410, 299.0175, 271.0246, 243.0319 |
| 95 | 31.50 | Tiliroside or regioisomeric derivative (I) | C_30_H_26_O_13_ | 593.1295 | 593.1313 | 285.0410, 284.0307, 255.0279, 227.0363, 145.0283 |
| 96 | 31.76 | Tiliroside or regioisomeric derivative (II) | C_30_H_26_O_13_ | 593.1295 | 593.1260 | 285.0373, 284.0307, 255.0279, 227.0331, 145.0283 |
| 97 | 31.96 | Tiliroside or regioisomeric derivative (III) | C_30_H_26_O_13_ | 593.1295 | 593.1313 | 285.0373, 284.0307, 255.0279, 227.0331, 145.0283 |
| 98 | 32.14 | Tiliroside or regioisomeric derivative (IV) | C_30_H_26_O_13_ | 593.1295 | 593.1313 | 284.0410, 284.0344, 255.0314, 227.0363, 145.0309 |
| 99 | 32.14 | Terpenmacrophylla derivative (I) | C_54_H_88_O_25_ | 1135.5536 | 1135.5557 | 1090.5420, 989.4876, 927.4948, 781.4385, 765.4390, 721.4244, 619.3831, 575.3585, 487.3405, 443.3503, 423.3264, 284.0307, 113.0241 |
| 100 | 32.23 | Quercetin 3,3'-dimethyl ether or regioisomeric derivative (I) | C_17_H_14_O_7_ | 329.0661 | 329.0637 | 299.0213, 271.0246, 243.0319 |
| 101 | 32.38 | Terpenmacrophylla derivative (II) | C_61_H_84_O_20_ | 1135.5478 | 1135.5483 | 1089.5388, 943.4930, 927.4948, 781.4385, 765.4450, 659.4168, 575.3585, 513.3544, 443.3181, 131.0344, 113.0241 |
| 102 | 33.76 | Terpengustifol derivative (I) | C_60_H_98_O_29_ | 1281.6116 | 1281.6088 | 1073.5590, 911.4947, 893.4895, 765.4450, 749.4493, 731.4337, 603.3909, 585.3793, 471.3475 |
| 103 | 35.04 | Kaempferol-*O*-rhamnosyl-*O*-hexosyl-*O*-hexosyl-*O*-rhamnoside (II) | C_43_H_54_O_21_ | 905.3079 | 905.3086 | 759.2529, 575.1436, 430.0901, 283.0259, 255.0279, 227.0298 |
| 104 | 35.04 | Quercetin 3,3'-dimethyl ether or regioisomeric derivative (II) | C_17_H_14_O_7_ | 329.0661 | 329.0667 | 299.0175, 271.0246, 243.0319, 199.0368 |
| 105 | 35.24 | Terpengustifol derivative (II) | C_59_H_96_O_28_ | 1251.601 | 1251.5972 | 1205.5990, 1043.5485, 749.4493, 603.3909, 585.3793, 471.3475 |
| 106 | 36.76 | Terpengustifol derivative (III) | C_54_H_88_O_24_ | 1119.5587 | 1119.5551 | 911.5013, 765.4390, 749.4493, 731.4337, 603.3909, 585.3793, 471.3475, 113.0241 |
| 107 | 37.80 | Terpenmacrophylla derivative (III) | C_31_H_50_O_8_ | 549.3427 | 549.3394 | 485.3255, 453.3008, 443.3135, 421.3095 |
| 108 | 38.40 | Quercetin 3,3'-dimethyl ether or regioisomeric derivative (III) | C_17_H_14_O_7_ | 329.0661 | 329.0637 | 299.0175, 271.0246, 243.0285, 215.0330 |
| 109 | 39.60 | Terpenmacrophylla derivative (IV) | C_30_H_48_O_6_ | 503.3373 | 503.3354 | 485.3255, 439.3232, 421.3095 |
| 110 | 40.01 | Terpenmacrophylla derivative (V) | C_30_H_48_O_6_ | 503.3373 | 503.3354 | 485.3255, 457.3306, 441.3368, 422.3151 |
| 111 | 41.38 | Terpenmacrophylla derivative (VI) | C_60_H_98_O_28_ | 1265.6166 | 1265.6174 | 1221.6125, 1057.5692, 895.5095, 877.4889, 749.4434, 733.4509, 715.4426, 587.3959, 569.3865, 455.3508 |
| 112 | 41.79 | Terpenmacrophylla derivative (VII) | C_54_H_88_O_23_ | 1103.5638 | 1103.5663 | 895.5030, 748.4434, 733.4509, 715.4426, 587.3959, 569.3865, 469.3707, 455.3508 |
| 113 | 42.42 | Terpengustifol derivative (IV) | C_68_H_108_O_30_ | 1403.6847 | 1403.6871 | 749.4434, 603.3909, 585.3793, 471.3475 |
| 114 | 42.96 | Terpenmacrophylla derivative (VIII) | C_54_H_88_O_23_ | 1103.5638 | 1103.5591 | 895.5030, 733.4568, 715.4426, 587.3959, 569.3813, 569.3813, 469.3707, 455.3508 |
| 115 | 43.48 | Terpenmacrophylla derivative (IX) | C_64_H_102_O_27_ | 1301.6530 | 1301.6484 | 1255.6525, 1093.6046, 1075.5796, 927.4948, 909.4764, 781.4385, 765.4450, 747.4282, 703.4377, 601.3791, 619.3831, 487.3453, 469.3281, 199.0954 |
| 116 | 44.09 | Terpenmacrophylla derivative (X) | C_65_H_104_O_28_ | 1331.6636 | 1331.6604 | 1285.6497, 745.4574, 649.3911, 605.3684, 539.3694, 487.3405, 443.3503, 183.1023, 165.0914, 139.1119 |
| 117 | 44.26 | Terpenmacrophylla derivative (XI) | C_64_H_102_O_27_ | 1301.6530 | 1301.6484 | 1255.6448, 909.4896, 765.4450, 619.3831, 575.3585, 487.3405, 443.3181, 427.3203, 183.1023, 165.0914, 139.1119, 113.0589 |
| 118 | 44.61 | Terpenmacrophylla derivative (XII) | C_70_H_110_O_30_ | 1429.7004 | 1429.7013 | 1387.6901, 1268.6442, 1101.5559, 807.4490, 747.4341, 685.4321, 661.3991, 601.3737, 529.3499, 469.3328 |
| 119 | 46.12 | Terpenmacrophylla derivative (XIII) | C_58_H_92_O_23_ | 1155.5951 | 1155.5973 | 1109.5941, 751.4254, 689.3864, 605.3684, 561.3412, 543.3312, 429.2971, 183.1023, 165.0914 |
| 120 | 46.28 | Terpenmacrophylla derivative (XIV) | C_30_H_48_O_5_ | 487.3423 | 487.3405 | 469.3328, 423.3264 |
| 121 | 46.48 | Terpenmacrophylla derivative (XV) | C_31_H_50_O_5_ | 533.3478 | 533.3454 | 487,3405 |
| 122 | 46.59 | Terpenmacrophylla derivative (XVI) | C_61_H_102_O_29_ | 1297.6429 | 1297.6454 | 1255.6448, 969.4987, 952.4978, 891.4652, 855.4396, 807.4490, 747.4341, 703.4377, 661.3997, 601.3737, 529.3499, 469.3328, 183.0994 |
| 123 | 46.74 | Terpenmacrophylla derivative (XVII) | C_31_H_50_O_5_ | 533.3478 | 533.3454 | 487,3405 |
| 124 | 46.75 | Terpengustifol A | C_69_H_110_O_29_ | 1401.7055 | 1401.7061 | 1309.5623, 1240.6635, 1073.5447, 749.4434, 603.3801, 585.3740, 471.3475 |
| 125 | 46.98 | Terpengustifol derivative (V) | C_53_H_88_O_22_ | 1075.5689 | 1075.5652 | 1030.5615, 705.4567, 559.3956, 487.3405, 471.3475 |
| 126 | 47.33 | Terpenmacrophylla derivative (XVIII) | C_31_H_50_O_5_ | 533.3478 | 533.3454 | 487,3405 |
| 127 | 47.33 | Terpenmacrophylla derivative (XIX) | C_30_H_48_O_5_ | 487.3423 | 487.3405 | 469.3328, 423.3264 |
| 128 | 47.51 | Terpenmacrophylla derivative (XX) | C_57_H_90_O_20_ | 1093.5947 | 1093.5957 | 855.4332, 765.4450, 721.4127, 703.4377, 619.3831, 575.3585, 557.3812, 487.3405, 443.3503, 183.1023, 165.0886, 139.1119 |
| 129 | 47.68 | Terpenmacrophylla derivative (XXI) | C_31_H_50_O_5_ | 533.3478 | 533.3454 | 487,3405 |
| 130 | 47.81 | Terpenmacrophylla derivative (XXII) | C_64_H_102_O_27_ | 1301.6530 | 1301.6563 | 1255.6448, 861.4981, 765.4510, 715.4368, 633.3986, 615.3966, 513.3594, 487.3405, 381.3171, 165.0914, 113.0264 |
| 131 | 48.11 | Terpengustifol derivative (VI) | C_69_H_110_O_30_ | 1417.7004 | 1417.7012 | 1371.6956, 1169.6088, 915.5445, 749.4434, 731.4337, 637.4484, 603.3855, 585.3740, 471.3427 |
| 132 | 48.11 | Terpenmacrophylla derivative (XXIII) | C_60_H_96_O_10_ | 975.6925 | 975.6884 | 487.3405, 469.3328, 423.3264 |
| 133 | 48.37 | Terpenmacrophylla derivative (XXIV) | C_58_H_92_O_22_ | 1139.6002 | 1139.5957 | 931.5399, 861.4981, 557.3812, 513.3594, 381.3171, 183.1023, 165.0914, 113.0589 |
| 134 | 48.47 | Terpenmacrophylla derivative (XXV) | C_31_H_50_O_5_ | 533.3478 | 533.3454 | 487,3405 |
| 135 | 48.63 | Terpenmacrophylla derivative (XXVI) | C_64_H_102_O_27_ | 1301.6530 | 1301.6484 | 1255.6448, 1093.5974, 931.5466, 785.4826, 765.4390, 747.4341, 619.3831, 575.3533, 487.3405, 183.1023, 165.0914, 113.0241 |
| 136 | 48.89 | Terpenmacrophylla derivative (XXVII) | C_59_H_94_O_23_ | 1169.6108 | 1169.6088 | 1139.6031, 1093.5902, 931.5466, 861.4981, 765.4450, 715.4450, 557.3864, 513.3594, 381.3128, 165.0914 |
| 137 | 49.45 | Terpenmacrophylla derivative (XXVIII) | C_69_H_108_O_28_ | 1371.6949 | 1371.6956 | 1210.6437, 1169.6088, 1139.5957, 1107.5345, 861.4981, 765.4510, 715.4368, 619.3885, 557.3812, 529.3529, 513.3594, 487.3405, 397.3127, 183.1023, 165.0914, 139.1119 |
| 138 | 50.20 | Terpenmacrophylla derivative (XXIX) | C_62_H_98_O_24_ | 1225.637 | 1225.6344 | 1169.6013, 1139.5957, 1093.5974, 931.5332, 785.4765, 765.4450, 747.4341, 619.3831, 513.3594, 487.3405, 473.3237, 381.3214, 183.1023, 139.1119 |
| 139 | 50.77 | Terpengustifol derivative (VII) | C_64_H_102_O_26_ | 1285.6581 | 1285.6575 | 1239.6628, 1077.5948, 893.4960, 765.4390, 749.4493, 731.4337, 637.4484, 603.3909, 485.3793, 471.3474, 453.3333, 183.1023 |
| 140 | 51.70 | Terpengustifol derivative (VIII) | C_58_H_92_O_21_ | 1123.6053 | 1123.6033 | 1077.6021, 911.5013, 765.4390, 749.4493, 731.4337, 637.4484, 603.3855, 585.3793, 471.3475, 453.3333, 183.1023 |
| 141 | 51.84 | Terpengustifol derivative (IX) | C_63_H_100_O_24_ | 1239.6526 | 1239.6552 | 1093.5974, 985.4625, 823.4091, 749.4493, 603.3909, 585.3793, 471.3475, 453.3333, 183.1023 |
| 142 | 53.85 | Terpenmacrophylla aglycones | C_30_H_48_O_5_ | 487.3423 | 487.3405 | 443.3457, 427.3203 |
| 143 – 148 | 56.78, 57.06, 58.41, 58.94, 59.19, 59.47 | Terpengustifol aglycones | C_30_H_48_O_4_ | 471.3474 | 471.3475 | 393,3144 |
| 149 – 151 | 70.72, 71.07, 71.54 | Ursolic and oleanolic acids of coumaroyl | C_39_H_54_O_6_ | 617.3842 | 617.3828 | 145.0283, 117.0351 |
| 152 – 155 | 71.80, 72.97, 73.16, 74.71 | Acetylated triterpenoids | C_39_H_54_O_6_ | 617.3842 | 617.3828 | 497.3265, 453.3380, 145.0283, 117.0327 |
| 156 – 161 | 78.41, 78.68, 79.01, 79.19, 79.55, 79.91 | *cis-* and *trans-* forms of caffeoyltriterpenic acid | C_39_H_54_O_6_ | 617.3842 | 617.3828 | 161.0248, 133.0286 |
| 162 - 165 | 84.58, 85.00, 85.32, 85.63 | *cis*- and *trans-* forms of 3-*O*-*p*-hydroxycinnamoyl ursolic or oleanolic acid | C_39_H_54_O_5_ | 601.3893 | 601.3898 | 437.3416, 145.0283, 117.0327 |


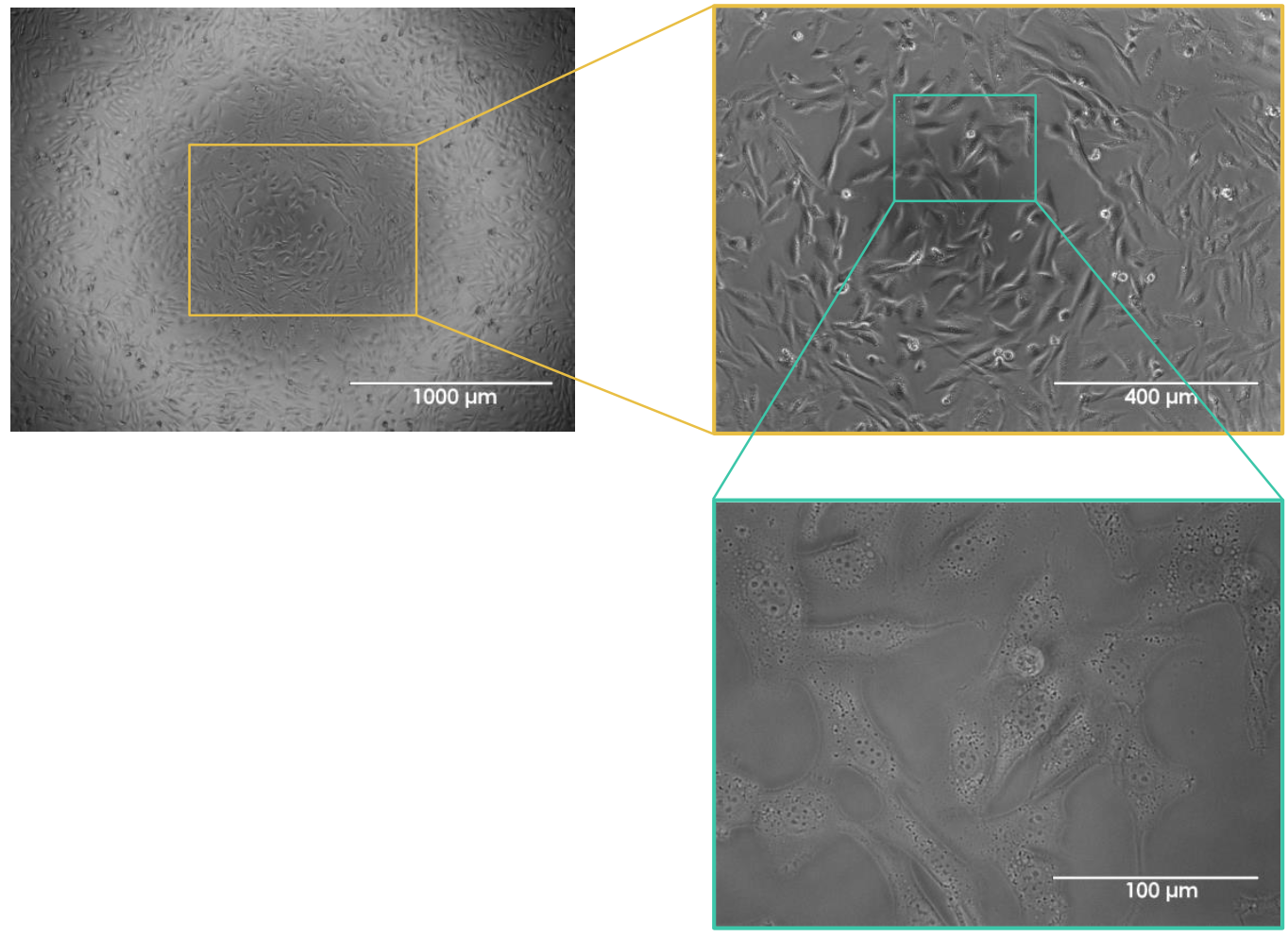


**Fig. S1.** NIH/3T3 cells observed at different magnifications, from left to right and top to bottom: x4, x10 and x40.


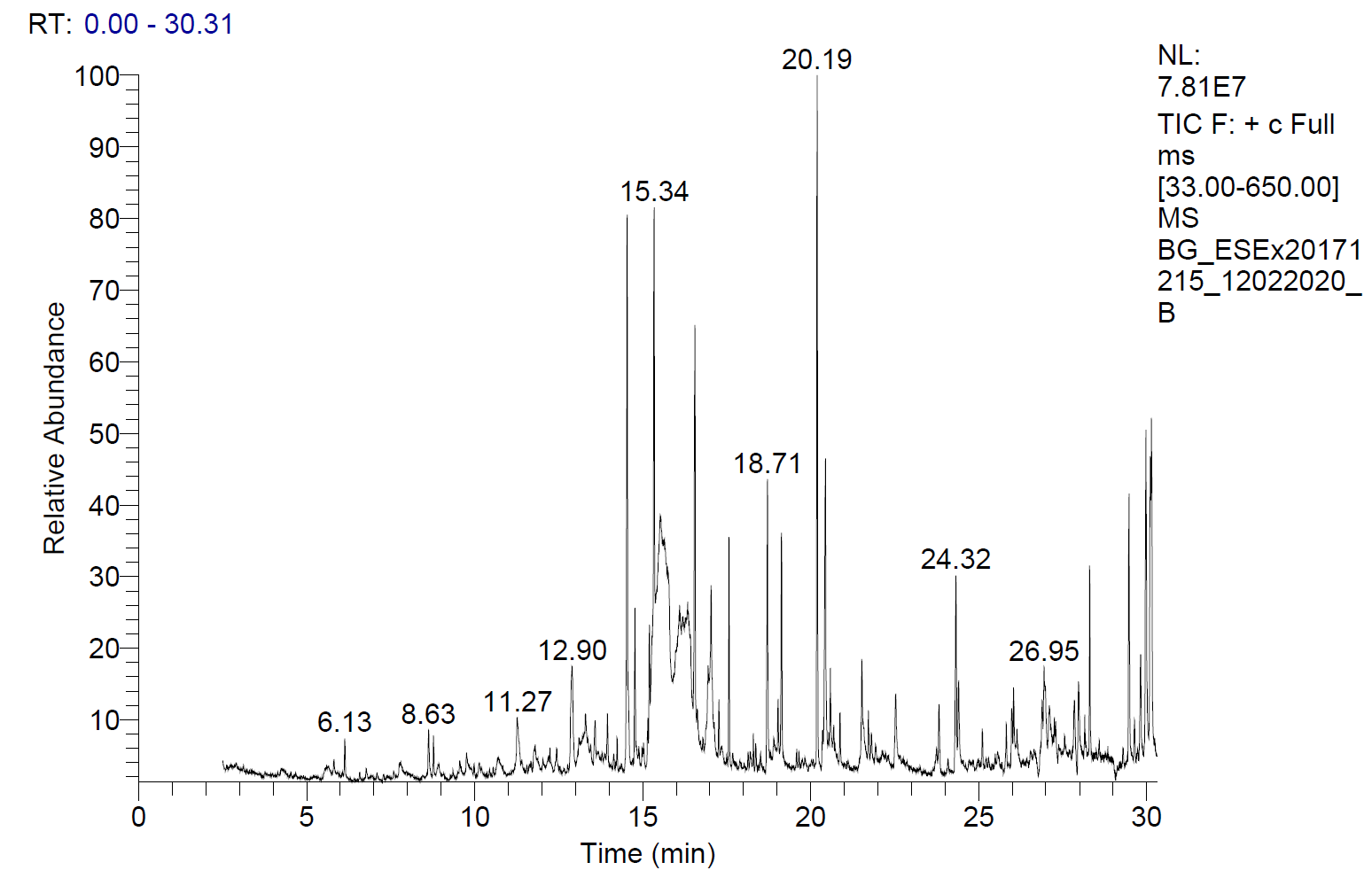


**Fig. S2.** Total ion chromatogram (TIC) of EE obtained by GC-MS.


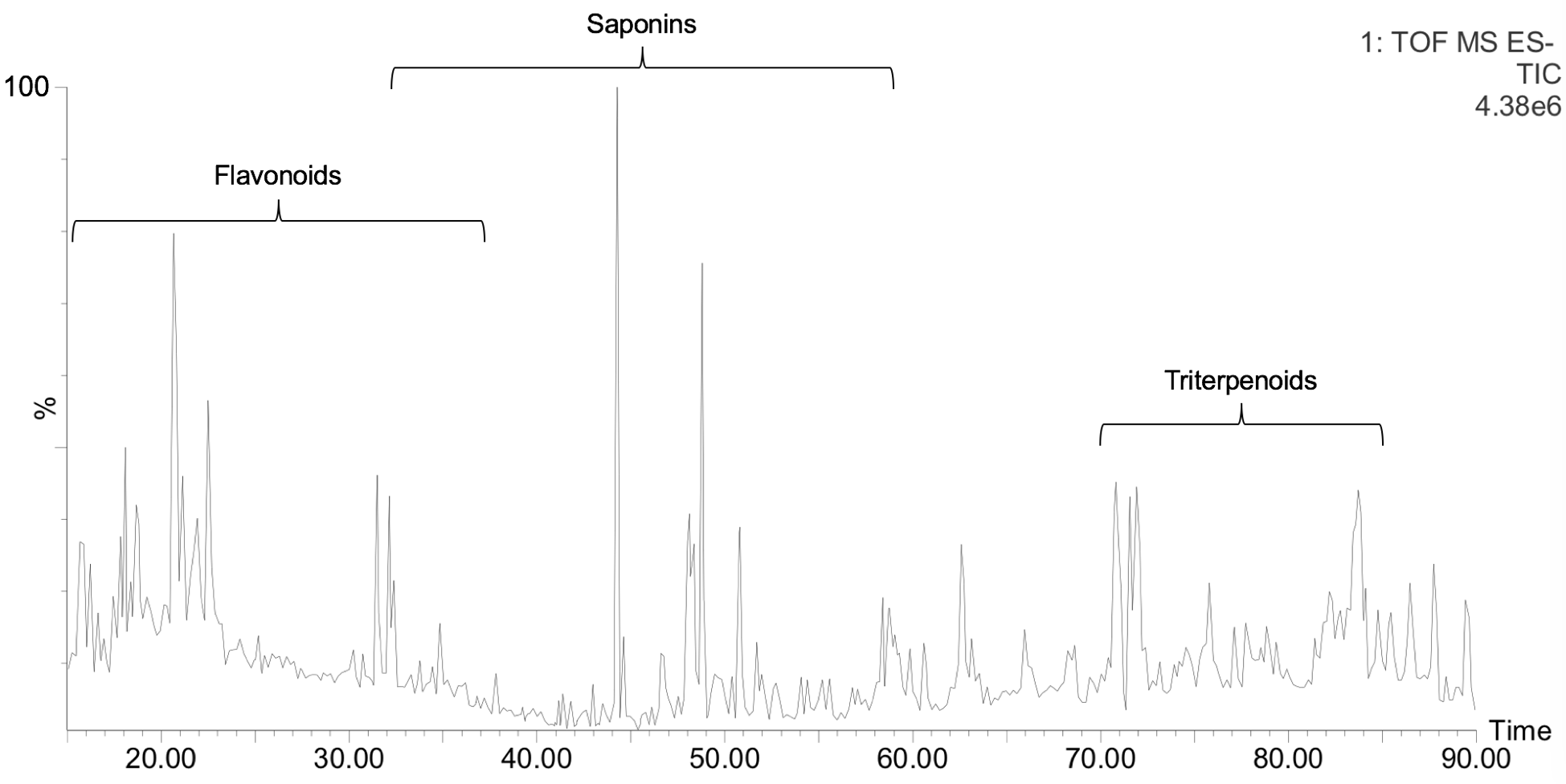


**Fig. S3.** Total ion chromatogram (TIC), in negative ionization mode, of EE.

**
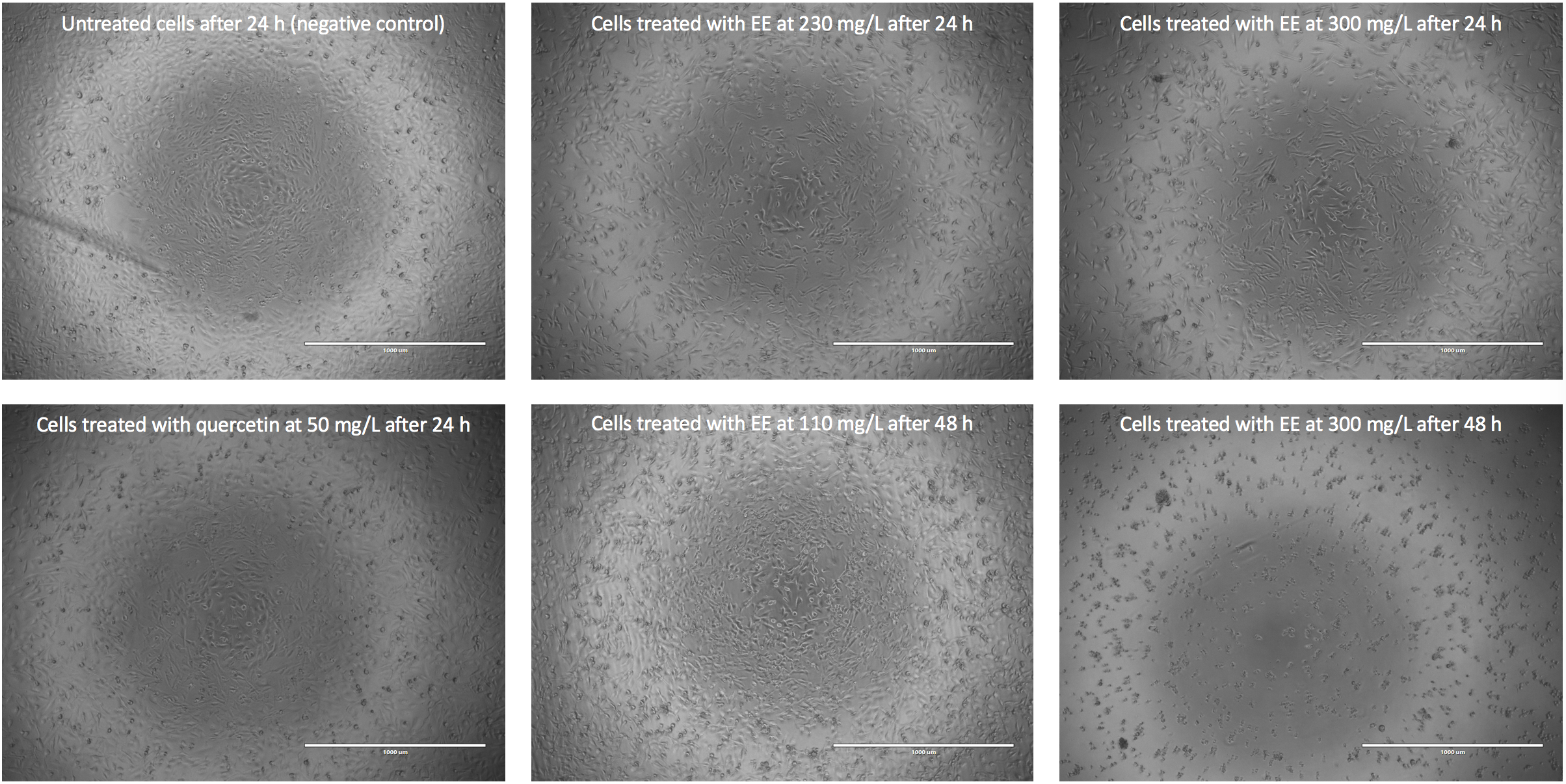
**

**Fig. S4.** Micrographs of NIH/3T3 cells viability after 24 h (negative control, EE at 230 and 300 mg/L and quercetin at 50 mg/L) and 48 h (EE at 110 and 300 mg/L) of treatment.

**Table S2**

Antioxidant capacity of an EE prepared from leaves collected in year N+1^1^.

|  | **TPC**  **(GAE/g)** | **DPPH**  **(mmol TE/g)** | **ABTS**  **(mmol TE/g)** | **ORAC value**  **(mmol TE/g)** | ***In cellulo* antioxidant capacity – ROS relative intensity**  **(%)** | | | | |
| --- | --- | --- | --- | --- | --- | --- | --- | --- | --- |
| EE (year N+1) | 79.69 ± 4.35 | 0.23 ± 0.03 | 0.70 ± 0.07 | 1.92 ± 0.35 | 69.5 ± 5.5  *at 50 mg/L* | 53.0 ± 9.1  *at 110 mg/L* | 60.5 ± 3.6  *at 170 mg/L* | 55.0 ± 5.4  *at 230 mg/L* | 52.4 ± 6.6  *at 300 mg/L* |

^1^ Leaves were collected from the same shrubs as those collected previously in year N. The ethanolic extraction was prepared according to the method described in section 2.3.
